# Trends in Use of Low-Value Care in Traditional Fee-for-Service Medicare and Medicare Advantage

**DOI:** 10.1001/jamanetworkopen.2021.1762

**Published:** 2021-03-17

**Authors:** Sungchul Park, Jeah Jung, Robert E. Burke, Eric B. Larson

**Affiliations:** 1 Department of Health Management and Policy, Dornsife School of Public Health, Drexel University, Philadelphia, Pennsylvania; 2 Department of Health Policy and Administration, College of Health and Human Development, Pennsylvania State University, University Park; 3 Center for Health Equity Research and Promotion, Corporal Michael J. Crescenz VA Medical Center, Philadelphia, Pennsylvania; 4 Division of General Internal Medicine, Department of Medicine, Perelman School of Medicine, University of Pennsylvania, Philadelphia; 5 Kaiser Permanente Washington Health Research Institute, Seattle

## Abstract

**Importance:**

Decreasing use of low-value care is a major goal for Medicare given the potential to decrease costs and harms. Compared with traditional fee-for-service Medicare (TM), Medicare Advantage (MA) is more strongly financially incentivized to decrease use of low-value care.

**Objectives:**

To compare use of low-value care among individuals enrolled in TM and those enrolled in MA overall and to examine trends in use of low-value care in both programs from 2006 to 2015.

**Design, Setting, and Participants:**

This cross-sectional study analyzed individuals enrolled in TM and MA using data from the 2006 to 2015 Medical Expenditure Panel Survey. To account for differences in characteristics between individuals enrolled in TM and those enrolled in MA, a propensity score–based approach was used. Data were analyzed from August 2020 through January 2021.

**Exposures:**

Being enrolled in MA or TM.

**Main Outcomes and Measures:**

Binary measures of use were collected for 13 low-value services in 4 categories (ie, [1] cancer screening: cervical, colorectal, and prostate cancer screening in older adults; [2] antibiotic use: antibiotic for acute upper respiratory infection and antibiotic for influenza; [3] medication: anxiolytic, sedative, or hypnotic in an adult older than 65 years; benzodiazepine for depression; opioid for headache; opioid for back pain; and nonsteroidal anti-inflammatory drug [NSAID] for hypertension, heart failure, or chronic kidney disease; and [4] imaging: magnetic resonance imaging [MRI] or computed tomography [CT] for back pain, radiograph for back pain, and MRI or CT for headache) and 4 low-value composites corresponding to the categories (ie, cancer screening composite, antibiotic use composite, medication composite, and imaging composite).

**Results:**

Among 11 677 individuals enrolled in TM and 5164 individuals enrolled in MA, 9429 (56.0%) were women and the mean (SD) age was 74.5 (6.3) years. Of 13 low-value services and 4 low-value composites, statistically significant differences were found in 2 measures. For the low-value medication composite, 2054 of 11 636 eligible individuals enrolled in TM (adjusted mean, 17.6%; 95% CI, 16.8%-18.3%) received the care, and 981 of 5141 eligible individuals enrolled in MA (adjusted mean, 19.7%; 95% CI, 18.3%-21.2%) received the care, for a rate of use that was significantly higher among individuals enrolled in MA, by 2.2 percentage points (95% CI, 0.5-3.8 percentage points; *P* = .02). For the NSAID use for hypertension, heart failure, or kidney disease metric, 807 of 7832 individuals enrolled in TM (adjusted mean, 10.0%; 95% CI, 9.2%-10.8%) received the care, and 447 of 3566 individuals enrolled in MA (adjusted mean, 12.9%; 95% CI, 19.7%-27.1%) received the care, for a rate of use that was significantly higher among individuals enrolled in MA, by 2.9 percentage points (95% CI, 1.3-4.6 percentage points; *P* = .001). Overall, there were no decreases in use of low-value care in TM or MA over time.

**Conclusions and Relevance:**

This cross-sectional study found that use of low-value care was similarly prevalent in MA and TM, suggesting that MA enrollment was not associated with decreased provision of low-value care compared with TM.

## Key Points

**Question:**

Were there differences in use of low-value care between individuals enrolled in traditional fee-for-service Medicare (TM) and those enrolled in Medicare Advantage (MA) from 2006 to 2015?

**Findings:**

In this cross-sectional study of 11 677 individuals enrolled in TM and 5164 individuals enrolled in MA, there were no significant differences between individuals enrolled in TM and those enrolled in MA in most measures of use of low-value care. There was no significant decrease in most measures of use of low-value care among individuals enrolled in TM or MA over time.

**Meaning:**

Use of low-value care is prevalent in TM and MA, suggesting that managed care enrollment is not associated with decreased use of low-value care.

## Teaser

This cross-sectional study compares use of low-value care among individuals enrolled in traditional Medicare and those enrolled in Medicare Advantage and examines trends in use from 2006 to 2015.

## Introduction

Low-value care is defined as a service that provides little to no clinical benefit but incurs health care costs.^1^ A significant share of health care costs in the US is of low value, estimated to account for 0.6% to 2.7% of annual health care spending^2^ or $75.7 billion to $101.2 billion annually.^3^ In some cases, low-value care can be associated with harmful patient outcomes and further unnecessary testing or treatment. Thus, decreasing use of low-value care has potential for cost savings and harm reduction in the US health care system.

Use of low-value care is especially prevalent among older adults in the US.^2,4,5,6,7,8,9,10^ A 2014 study^2^ found that among 26 low-value services in 6 categories (ie, cancer screening, diagnostic and preventive testing, preoperative testing, imaging, cardiovascular testing and procedures, and other surgical procedures), 24% to 41% of individuals enrolled in traditional fee-for-service Medicare (TM) received at least 1 low-value service in 2009. The rate of use of low-value care varied across services, ranging from 0.1% for electroencephalography for headache to 12.4% for imaging for low-risk low back pain. Among the 6 categories of low-value care, the most prevalent were imaging, which accounted for 43% of low-value care, and cancer screening, which accounted for 31% of low-value care.

Although most individuals enrolled in Medicare receive care through TM, 36% of individuals enrolled in Medicare were enrolled in Medicare Advantage (MA), known as Medicare managed care, in 2020.^11^ Understanding whether individuals enrolled in TM and those enrolled in MA had different rates of use of low-value care may be of high policy relevance because MA plans are financially incentivized to use lower-cost care through capitated payments. These plans can also implement network and prior authorization restrictions to control health care use. Furthermore, MA plans can offer supplemental benefits to address the needs of individuals with chronic illness.^12,13^ Indeed, MA tends to deliver care more efficiently than TM by increasing use of primary care visits^14,15^ and decreasing use of unnecessary care or intensity of care.^16,17,18,19,20^ However, to our knowledge, evidence on use of low-value care in MA is limited. A 2017 study^5^ examined use of low-value care in MA but was limited to using data from a single insurer. Evidence suggests that low-value services among individuals enrolled in MA varied widely in frequency and cost, with spending on low-value care in 2014 totaling $100.8 million for individuals enrolled in MA.^5^

Another important policy question is how use of low-value care in TM and MA has changed over time. Since 2012, several initiatives, including Choosing Wisely,^1^ have been launched to decrease use of low-value care. Evidence suggests that Choosing Wisely was associated with modest decreases in use of low-value care in TM and MA.^5,6,21,22^ Concurrently, new delivery models have been implemented to decrease use of unnecessary care. Evidence suggests that Medicare accountable care organization (ACO) programs were associated with decreases in use of low-value care, but the magnitude of the decreases was small.^22^ However, it remains unknown whether this trend occurred in both TM and MA.

We addressed these knowledge gaps regarding use of low-value care in TM and MA. First, we compared use of low-value care between individuals enrolled in TM and those enrolled in MA. Second, we examined trends in use of low-value care in the programs from 2006 to 2015.

## Methods

This cross-sectional study was exempted from review and informed consent by the institutional review board of Drexel University because the data were deidentified and publicly available. Reporting followed the Strengthening the Reporting of Observational Studies in Epidemiology (STROBE) reporting guideline.

### Data and Study Sample

We used data from the 2006 to 2015 Medical Expenditure Panel Survey (MEPS). The MEPS is a nationally representative survey of the US noninstitutionalized civilian population. This survey annually collects information on respondents’ demographic and socioeconomic characteristics, health status, and health care use. The data are collected in person via 3 rounds per year.

Specifically, we used the following 6 data sets from the MEPS: the full-year consolidated data files, longitudinal files, medical condition files, outpatient visit files, office-based medical clinic visit files, and prescribed medicine files. Compared with claims data, the MEPS has several strengths for comparing use of low-value care between individuals enrolled in TM and MA. First, the MEPS offers service-specific data for those enrolled in MA, which allowed us to identify these individuals’ use of low-value care. By comparison, claims data for this population is limited. Second, the MEPS provides comprehensive information on socioeconomic and health status, which are associated with rates of health services use but are imperfectly captured in claims data. Third, clinician coding patterns may be associated with differences in health risks as constructed from claims data; studies from 2014^23^ and 2020^24^ suggest differences in coding between MA and TM. However, the MEPS offers self-reported health status, which allowed us to control for a measure of health that is comparable across TM and MA populations.

We included individuals enrolled in Medicare for the entire year sampled and excluded those who were younger than 65 years, were dual eligible for Medicare and Medicaid, and switched between TM and MA within the year. The MEPS asked respondents whether they enrolled in MA or TM in each round of data collection (conducted 3 times every year). We defined individuals as enrolled in TM if they were continuously enrolled in the program throughout the 3 rounds in the year, and we identified individuals as enrolled in MA if they were continuously enrolled in the program throughout the 3 rounds.

### Measurement of Low-Value Care

Following studies from 2016^25^ and 2019^26^ that identified low-value care using the MEPS, we identified 13 low-value services in 4 categories. Use of low-value care was measured as a binary outcome. For each measure,^27,28,29,30,31,32,33,34,35,36^ we identified individuals who were eligible for the measure (ie, the denominator) and then determined whether those individuals received the particular low-value service (ie, the numerator) (Table 1). We excluded individuals with some conditions associated with clinical red flags, because use of some care for particular conditions may not be deemed to be low value. Additionally, we constructed a composite measure for each of the 4 categories of low-value care; for these categories, use was defined as the use of any low-value service in the category.^27,28,29,30,31,32,33,34,35,36^

**Table 1.** Measures for Low-Value Care

Measure or composite	Source	MEPS data source	Measure (ie, numerator)	Eligible population (ie, denominator)
Low-value cancer screening composite				
Cervical cancer screening	Vesco et al,^27^ 2011	Self-report	Papanicolaou test in the given survey year	Women aged >65 y without a diagnosis of cervical cancer or other genital cancer found among women in the prior year^a^
Colorectal cancer screening	Whitlock et al,^28^ 2008	Self-report	Colorectal cancer screening (ie, colonoscopy, sigmoidoscopy, or hemoccult test) in the given survey year	Individuals aged >75 y without a diagnosis of colon cancer in the prior year^a^
Prostate cancer screening	Lin et al,^29^ 2011	Self-report	Prostate-specific antigen test in the given survey year	Men aged >75 y without a diagnosis of prostate cancer in the prior year^a^
Low-value antibiotic use composite				
Antibiotic for acute upper respiratory infection	Cooper et al,^30^ 2001	Prescribed medicine	Antibiotic prescription during visit	Individuals with a primary diagnosis of acute upper respiratory infection without a diagnosis of bacterial infection, chronic obstructive pulmonary disease, or cancer in the given survey year (as competing diagnosis for acute upper respiratory infection)^b^
Antibiotic for influenza	Cooper et al,^30^ 2001	Prescribed medicine	Antibiotic prescription during visit	Individuals with a primary diagnosis of influenza without a diagnosis of bacterial infection, chronic obstructive pulmonary disease, or cancer in the given survey year^b^
Low-value medication composite				
Anxiolytic, sedative, or hypnotic	American Geriatrics Society 2012 Beers Criteria Update Expert Panel,^31^ 2012	Prescribed medicine	Anxiolytic, sedative, or hypnotic prescription during visit	Individuals aged >65 y
Benzodiazepine for depression	Trangle et al,^32^ 2016	Prescribed medicine	Benzodiazepine prescription during visit	Individuals with a diagnosis of depression
Opioid for headache	American Academy of Neurology,^33^ 2013	Prescribed medicine	Opioid prescription during visit	Individuals with a diagnosis of headache but no diagnosis of pregnancy, cancer, or epilepsy in the given survey year^c^
Opioid for back pain	American Society of Anesthesiologists,^34^ 2014	Prescribed medicine	Opioid prescription during visit	Individuals with a diagnosis of back pain but no diagnosis of fever or cancer in the given survey year^d^
NSAID for hypertension, heart failure, or kidney disease	American Society of Anesthesiologists,^34^ 2014	Prescribed medicine	NSAID prescription during visit	Individuals with a diagnosis of hypertension, heart failure, or kidney disease in the given survey year
Low-value imaging composite				
MRI or CT for back pain	Chou et al,^35^ 2009	Outpatient^e^	MRI or CT scan during visit	Individuals with a diagnosis of back pain but no diagnosis of fever or cancer in the given survey year^d^
Radiograph for back pain	Chou et al,^35^ 2009	Outpatient^e^	Radiograph during visit	Individuals with a diagnosis of back pain but no diagnosis of fever or cancer in the given survey year^d^
MRI or CT for headache	American College of Radiology,^36^ 2012	Outpatient^e^	MRI or CT scan during visit	Individuals with a diagnosis of headache but no diagnosis of pregnancy, cancer, or epilepsy in the given survey year^c^

Abbreviations: CT, computed tomography; MEPS, Medical Expenditure Panel Survey; MRI, magnetic resonance imaging; NSAID, nonsteroidal anti-inflammatory drug.

^a^ History of cancer in prior years could not be identified, owing to lack of data availability. As an alternative, MEPS longitudinal files were used to identify individuals diagnosed with each type of cancer in the prior year.

^b^ Vaginitis and HIV could not be included as competing diagnoses because they could not be identified based on Clinical Classification Software or 3-digit *International Classification of Diseases, Ninth Revision, Clinical Modification* codes.

^c^ Not all conditions associated with clinical red flags (ie, HIV, neurologic symptoms [ie, weakness, sensory changes, and altered mental status], and head trauma) could be excluded, because they could not be identified based on Clinical Classification Software or 3-digit *International Classification of Diseases, Ninth Revision, Clinical Modification* codes.

^d^ Not all conditions associated with clinical red flags (ie, weight loss, cachexia, neurologic symptoms, spinal fracture, myelopathy, and postlaminectomy) could be excluded, because they could not be identified based on Clinical Classification Software or 3-digit *International Classification of Diseases, Ninth Revision, Clinical Modification* codes.

^e^ Includes office-based medical clinic visit.

The cancer composite included 3 low-value cancer screenings: cervical, colorectal, and prostate cancer screening in older adults. We used self-reports to identify use of cancer screening in a given year. Using longitudinal files, we excluded individuals diagnosed with each type of cancer in the prior year. The treatment composite had 2 low-value antibiotic use measures: antibiotic for acute upper respiratory infection and antibiotic for influenza. The medication composite had 5 measures for use of low-value medication: use of an anxiolytic, a sedative, or a hypnotic in an adult older than 65 years; use of benzodiazepine for depression; use of opioid for headache; use of opioid for back pain; and use of nonsteroidal anti-inflammatory drug (NSAID) for hypertension, heart failure, or chronic kidney disease. For measures in the treatment and medication composites, we first used the medical condition files to identify individuals eligible for each measure and then used the prescribed medicine files to identify use of specific medications or services as an indication for each condition. Use of prescription drugs was identified via drug names or therapeutic drug classes. We identified therapeutic drug classes for each prescription using the National Drug Code directory, generic names, and the Multum Lexicon therapeutic classification database (Cerner Multum). The imaging composite included 3 measures capturing use of a low-value imaging test: magnetic resonance imaging (MRI) or computed tomography (CT) for back pain, radiograph for back pain, and MRI or CT for headache. Using medical condition files, we identified individuals eligible for each measure. Then, we used outpatient visit files and office-based clinic visit files to identify use of imaging during visit. For each measure in the treatment, medication, and imaging composites, we excluded individuals with competing diagnoses or with some conditions associated with clinical red flags (Table 1).

Following studies from 2016^25^ and 2019,^26^ we identified individuals enrolled in Medicare eligible for each measure using the Clinical Classification Software (CCS) for the *International Classification of Diseases, Ninth Revision, Clinical Modification* (*ICD-9-CM*).^37^ This software collapses diagnosis and procedure codes from the *ICD-9-CM* and then aggregates the codes into clinically meaningful categories that group similar conditions.

### Covariates

We included individual-level demographic, socioeconomic, and health status variables. These were age, sex, race/ethnicity, marital status, family income, US census region of residence, perceived health status, perceived mental health status, presence of any limitation, presence of cognitive limitation, presence of functional limitation, presence of social limitation, and 13 chronic conditions.

### Statistical Analysis

We compared unadjusted and adjusted sample characteristics between individuals enrolled in TM and those enrolled in MA. Studies from 2012 to 2019^38,39,40^ found that individuals enrolled in MA are healthier than those enrolled in TM, making a direct comparison between the 2 groups potentially biased. To address selective enrollment, we used a propensity score–based approach. Following a 2020 study,^14^ we computed the inverse probability of treatment weighting (IPTW) as a propensity for enrolling in MA based on the variables described. Enrollment in MA has grown rapidly over the past decade,^11^ suggesting potential differences in the composition of TM and MA populations over time. To account for changes in these compositions over time, we estimated IPTW separately for 5 periods (ie, 2006-2007, 2008-2009, 2010-2011, 2012-2013, and 2014-2015). We then determined whether IPTW-weighted samples were balanced on individual-level demographic, socioeconomic, and health status variables between TM and MA populations overall as well as within each period. We also determined whether the propensity score had an adequate overlap between TM and MA populations in each period.

To estimate differences in use of low-value care between individuals enrolled in TM and those enrolled in MA, we performed logit estimation after controlling for all covariates described previously, as well as MA enrollment, periods (ie, 2006-2007, 2008-2009, 2010-2011, 2012-2013, and 2014-2015), interaction terms associated with MA enrollment and periods, interaction terms associated with census regions and periods, and share of individuals enrolled in Medicare enrolled in MA plans by year and census region. We applied IPTW while conducting the analysis. From the regression results, we calculated the mean adjusted values of the outcomes for individuals enrolled in TM and MA while holding constant all other variables except the variable of interest, allowing us to compare the outcome of interest between individuals enrolled in TM and those enrolled in MA. We then examined the difference in these adjusted mean outcomes among individuals enrolled in MA compared with those enrolled in TM.

We also examined trends in use of low-value care among individuals enrolled in TM and MA using 4 low-value care composite measures. We conducted the same analysis as described previously and estimated the adjusted mean outcomes for individuals enrolled in TM and MA in each period. Because changes in data collection may be associated with changes in trends, we compared the numbers for individuals eligible for each measure across 5 periods. We also examined whether the sample size varied by year in association with sample fluctuations.

We conducted several sensitivity analyses to determine the robustness of our findings. First, because MEPS has smaller sample sizes than claims data do, which could bias toward the null, we conducted bootstrapping with 1000 iterations to measure robust CIs. Second, we conducted a heterogeneity analysis to identify whether prevalence of low-value care varied by socioeconomic and health status. We performed the analysis stratified by race/ethnicity, family income, and perceived health status.

For all analyses, we clustered standard errors within individuals because some individuals were included in the data over the course of multiple years. We used survey weights to adjust sample characteristics to be representative of the Medicare population. All *P* values were from 2-sided tests, and results were deemed significant at *P* < .05. Study data were analyzed using Stata statistical software version 16.1 (StataCorp) from August 2020 through January 2021.

## Results

Among 11 677 individuals enrolled in TM and 5164 individuals enrolled in MA, mean [SD] age was 74.5 [6.3] years and 9429 (56.0%) were women (Table 2). While there were several differences in weighted sample characteristics between TM and MA populations, differences in health status variables were marginal. In our analyses estimating IPTW, we found that MA enrollment was not associated with better health status (eTable 1 in the Supplement). These differences decreased after applying IPTW. Additionally, demographic, socioeconomic, and health status variables of individuals enrolled in TM and MA were similar in each of the 5 periods (eTable 2 in the Supplement). There was a substantial overlap in propensity scores between individuals enrolled in TM and those enrolled in MA in each of the 5 periods (eFigure in the Supplement).

**Table 2.** Characteristics of Individuals Enrolled in Medicare, 2006 to 2015

Characteristic	No. (%)		Weighted % (95% CI)				
			Without IPTW^a^			With IPTW	
	TM (n = 11 677)	MA (n = 5164)	TM	MA	TM		MA
Age, y							
65-69	3391 (29.0)	1450 (28.1)	27.8 (27.0-28.6)	27.4 (26.2-28.6)	27.8 (27.0-28.6)		28.2 (27.0-29.4)
70-74	2925 (25.0)	1369 (26.5)	25.6 (24.8-26.4)	25.9 (24.7-27.1)	25.7 (24.9-26.4)		25.3 (24.1-26.5)
75-79	2291 (19.6)	1074 (20.8)	20.0 (19.2-20.7)	20.5 (19.4-21.6)	20.1 (19.4-20.8)		20.3 (19.2-21.4)
≥80	3070 (26.3)	1271 (24.6)	26.6 (25.8-27.4)	26.2 (25-27.4)	26.4 (25.6-27.2)		26.2 (25.0-27.4)
Sex							
Men	5260 (45.0)	2152 (41.7)	45.4 (44.5-46.3)	42.0 (40.6-43.3)	44.4 (43.5-45.3)		44.7 (43.4-46.1)
Women	6417 (55.0)	3012 (58.3)	54.6 (53.7-55.5)	58.0 (56.6-59.3)	55.5 (54.6-56.4)		55.2 (53.9-56.6)
Race/ethnicity							
Non-Hispanic White	8973 (76.8)	3183 (61.6)	87.6 (87.1-88.2)	79.2 (78.1-80.3)	84.9 (84.3-85.6)		85.0 (84.0-86.0)
Hispanic	583 (5.0)	780 (15.1)	2.8 (2.5-3.1)	8.6 (7.8-9.4)	4.8 (4.4-5.2)		4.7 (4.1-5.3)
Non-Hispanic Black	1553 (13.3)	760 (14.7)	6.1 (5.7-6.6)	7.3 (6.6-8)	6.4 (6-6.9)		6.5 (5.8-7.2)
Non-Hispanic Asian	371 (3.2)	365 (7.1)	1.8 (1.5-2.0)	3.8 (3.3-4.3)	2.3 (2.1-2.6)		2.3 (1.9-2.7)
Other or multiple	197 (1.7)	76 (1.5)	1.7 (1.4-1.9)	1.1 (0.8-1.4)	1.5 (1.3-1.7)		1.5 (1.2-1.9)
Married	6736 (57.7)	2820 (54.6)	59.6 (58.7-60.5)	56.5 (55.1-57.8)	58.8 (57.9-59.7)		59.0 (57.6-60.3)
Family income, % of FPL							
<200	3759 (32.2)	1864 (36.1)	27.4 (26.6-28.2)	31.2 (29.9-32.5)	28.5 (27.6-29.3)		28.3 (27.1-29.5)
200-399	3530 (30.2)	1737 (33.6)	28.8 (27.9-29.6)	32.8 (31.5-34.1)	30.1 (29.3-30.9)		31.0 (29.8-32.3)
≥400	4388 (37.6)	1563 (30.3)	43.8 (42.9-44.7)	36.0 (34.7-37.3)	41.4 (40.5-42.3)		40.7 (39.4-42.0)
US Census region							
Northeast	1748 (15.0)	760 (14.7)	17.4 (16.7-18.1)	17.8 (16.8-18.9)	17.4 (16.7-18.0)		17.1 (16.1-18.1)
Midwest	3049 (26.1)	845 (16.4)	26.3 (25.5-27.1)	18.4 (17.3-19.5)	24.0 (23.3-24.8)		24.4 (23.3-25.6)
South	5072 (43.4)	1718 (33.3)	41.7 (40.8-42.6)	31.1 (29.9-32.4)	38.5 (37.6-39.3)		38.5 (37.1-39.8)
West	1808 (15.5)	1841 (35.7)	14.6 (14.0-15.2)	32.6 (31.4-33.9)	20.2 (19.4-20.9)		20.0 (19.0-21.1)
Perceived health status							
Excellent, very good, or good	9177 (78.6)	4069 (78.8)	80.3 (79.6-81.1)	81.8 (80.8-82.9)	80.8 (80.0-81.5)		81.1 (80.0-82.1)
Poor or fair	2469 (21.1)	1087 (21.0)	19.4 (18.7-20.2)	18.1 (17.0-19.1)	19.0 (18.3-19.7)		18.8 (17.7-19.9)
Perceived mental health status							
Excellent, very good, or good	10 461 (89.6)	4644 (89.9)	90.7 (90.2-91.2)	91.2 (90.4-92.0)	90.9 (90.4-91.4)		91.1 (90.3-91.9)
Poor or fair	1188 (10.2)	513 (9.9)	9.1 (8.6-9.6)	8.7 (7.9-9.5)	8.9 (8.4-9.4)		8.8 (8.0-9.6)
Limitation							
Any	3259 (27.9)	1398 (27.1)	27.0 (26.2-27.8)	26.4 (25.2-27.6)	26.7 (25.9-27.5)		26.3 (25.1-27.5)
Functional	1664 (14.3)	731 (14.2)	13.5 (12.9-14.1)	13.4 (12.4-14.3)	13.3 (12.7-14.0)		12.9 (12.0-13.8)
Cognitive	4963 (42.5)	2067 (40.0)	42.3 (41.4-43.2)	40.8 (39.4-42.1)	41.8 (40.9-42.6)		41.7 (40.3-43.0)
Social	1949 (16.7)	776 (15.0)	15.9 (15.2-16.6)	15.0 (14.1-16.0)	15.6 (14.9-16.2)		15.7 (14.7-16.7)
Chronic condition							
Angina	967 (8.3)	347 (6.7)	8.5 (8.0-9)	7.2 (6.5-8.0)	8.1 (7.6-8.6)		8.1 (7.3-8.8)
Arthritis	6877 (58.9)	3115 (60.3)	59.2 (58.3-60.1)	60.5 (59.2-61.8)	59.5 (58.6-60.4)		59.5 (58.2-60.9)
Asthma	1011 (8.7)	447 (8.7)	8.8 (8.2-9.3)	8.4 (7.6-9.1)	8.7 (8.2-9.2)		8.6 (7.8-9.3)
Coronary heart disease	2277 (19.5)	893 (17.3)	20.0 (19.2-20.7)	18.9 (17.8-19.9)	19.7 (18.9-20.4)		19.8 (18.7-20.8)
Diabetes	2652 (22.7)	1263 (24.5)	20.7 (20.0-21.5)	21.8 (20.7-22.9)	21.0 (20.2-21.7)		20.9 (19.8-22.0)
Emphysema	735 (6.3)	312 (6.0)	6.6 (6.2-7.1)	6.4 (5.7-7.0)	6.5 (6.1-7)		6.7 (6.0-7.4)
High cholesterol	7314 (62.6)	3313 (64.2)	62.8 (61.9-63.6)	64.8 (63.5-66.1)	63.2 (62.3-64.1)		63.5 (62.2-64.8)
High blood pressure	8121 (69.5)	3664 (71.0)	68.2 (67.4-69.1)	69.1 (67.9-70.4)	68.4 (67.6-69.3)		68.0 (66.8-69.3)
MI	1415 (12.1)	547 (10.6)	12.3 (11.8-12.9)	11.5 (10.7-12.4)	12.2 (11.6-12.8)		12.3 (11.4-13.2)
Stroke	3179 (27.2)	1273 (24.7)	28.9 (28.1-29.7)	27 (25.8-28.2)	28.3 (27.5-29.2)		28.1 (26.9-29.3)
Other heart condition	1401 (12.0)	600 (11.6)	11.7 (11.1-12.3)	11.8 (10.9-12.7)	11.7 (11.1-12.3)		11.3 (10.5-12.2)
Cancer	2626 (22.5)	954 (18.5)	24.9 (24.1-25.7)	21.3 (20.2-22.4)	23.8 (23-24.5)		24.2 (23-25.4)
AD and related dementia	458 (3.9)	150 (2.9)	3.6 (3.3-4)	2.9 (2.4-3.3)	3.4 (3.1-3.7)		3.3 (2.8-3.8)

Abbreviations: AD, Alzheimer disease; FPL, federal poverty level; IPTW, inverse probability of treatment weighting; MA, Medicare Advantage; MI, myocardial infarction; TM, traditional Medicare.

^a^ Sampling weights provided by the Medical Expenditure Panel Survey data were used.

Our IPTW-adjusted analyses found no significant differences between individuals enrolled in TM and those enrolled in MA in use of most of the measures of low-value care (Table 3). Statistically significant differences were found in 2 measures: low-value medication composite and NSAID use for hypertension, heart failure, or kidney disease. For the low-value medication composite, 2054 of 11 636 eligible individuals enrolled in TM (adjusted mean, 17.6%; 95% CI, 16.8%-18.3%) received the care, and 981 of 5141 eligible individuals enrolled in MA (adjusted mean, 19.7%; 95% CI, 18.3%-21.2%) received the care, for a rate of use that was statistically significantly higher among individuals enrolled in MA, by 2.2 percentage points (95% CI, 0.5-3.8 percentage points; *P* = .02). For NSAID use for hypertension, heart failure, or kidney disease, 807 of 7832 eligible individuals enrolled in TM (adjusted mean, 10.0%; 95% CI, 9.2%-10.8%) received the care, and 447 of 3566 eligible individuals enrolled in MA (adjusted mean, 12.9%; 95% CI, 19.7%-27.1%) received the care, for a rate of use that was statistically significantly higher among individuals enrolled in MA, by 2.9 percentage points (95% CI, 1.3-4.6 percentage points; *P* = .001). For other outcomes, use of low-value service was higher among individuals enrolled in MA than those enrolled in TM except for colorectal cancer screening, but no difference was statistically significant.

**Table 3.** Use of Low-Value Care, 2006 to 2015

Measure or composite	TM			MA			Difference, mean % (95% CI)	P value
	Eligible patients, No.	Patients receiving care, No.	Adjusted mean, % (95% CI)	Eligible patients, No.	Patients receiving care, No.	Adjusted mean, % (95% CI)		
Low-value cancer screening composite	8375	1577	18.1 (17.2-19.0)	3793	659	18.7 (17.1-20.3)	0.6 (−1.3 to 2.5)	.43
Cervical cancer screening	6307	852	12.6 (11.7-13.5)	2948	372	13.3 (11.7-14.9)	0.7 (**−**1.2 to 2.5)	.56
Colorectal cancer screening	4830	312	5.9 (5.2-6.6)	2100	110	5.1 (3.9-6.4)	−0.8 (**−**2.2 to 0.7)	.35
Prostate cancer screening	2068	565	27.9 (25.7-30.2)	845	231	28.8 (25.3-32.3)	0.9 (**−**3.3 to 5.1)	.55
Low-value antibiotic use composite	1942	620	31.1 (28.7-33.4)	799	232	33.2 (29.1-37.3)	2.1 (**−**2.5 to 6.8)	.19
Antibiotic for acute upper respiratory infection	1658	557	33.2 (30.6-35.8)	658	207	35.6 (31.0-40.1)	2.3 (**−**2.8 to 7.5)	.19
Antibiotic for influenza	339	67	17.4 (12.8-22.0)	167	27	17.7 (11.8-23.6)	0.3 (**−**7.5 to 8.1)	.54
Low-value medication composite	11 636	2054	17.6 (16.8-18.3)	5141	981	19.7 (18.3-21.2)	2.2 (0.5 to 3.8)	.02
Anxiolytic, sedative, or hypnotic	11 461	931	8.2 (7.6-8.7)	5064	422	8.9 (7.8-9.9)	0.7 (**−**0.5 to 1.9)	.34
Benzodiazepine for depression	1127	512	46.4 (43.0-49.8)	455	212	47.0 (41.9-52.1)	0.6 (**−**5.5 to 6.7)	.40
Opioid for headache	331	NA^a^	NA^a^	140	NA^a^	NA^a^	NA^a^	NA
Opioid for back pain	1847	264	14.0 (12.2-15.7)	762	122	14.6 (11.8-17.5)	0.7 (**−**2.7 to 4.0)	.43
NSAID for hypertension, heart failure, or kidney disease	7832	807	10.0 (9.2-10.8)	3566	447	12.9 (11.5-14.4)	2.9 (1.3 to 4.6)	.001
Low-value imaging composite	2094	457	22.0 (19.9-24.1)	870	174	23.4 (19.7-27.1)	1.4 (**−**2.9 to 5.7)	.34
MRI or CT for back pain	1847	224	12.3 (10.6-14.1)	762	83	12.5 (9.7-15.3)	0.1 (**−**3.1 to 3.4)	.57
Radiograph for back pain	1847	284	15.1 (13.1-17.0)	762	109	17.0 (13.4-20.6)	1.9 (**−**2.2 to 6.0)	.31
MRI or CT for headache	331	NA^a^	NA^a^	140	NA^a^	NA^a^	NA^a^	NA

Abbreviations: CT, computed tomography; MA, Medicare Advantage; MRI, magnetic resonance imaging; NA, not applicable; NSAID, nonsteroidal anti-inflammatory drug; TM, traditional Medicare.

^a^ There were fewer than 5 individuals with use of low-value care.

Odds ratios (ORs) from logit estimation of use of low-value care between individuals enrolled in TM and those enrolled in MA are presented in eTable 3 in the Supplement. In subgroup analyses, there were no differences in the cancer or treatment composites. Being a non-Hispanic White individual, being in good perceived health, and having an income greater than 400% of the federal poverty level were associated with higher rates of low-value medication use in the MA population, and being in poor health was associated with higher rates of low-value imaging, as measured by the imaging composite, in the MA population (eTable 4 in the Supplement).

Our analyses also found little evidence that use of low-value care among individuals enrolled in TM and MA decreased over time. Overall, there were no decreases in low-value composite measures in TM or MA over time except for the low-value cancer screening composite, which decreased in the 2 populations similarly over time (Figure). In other words, there was a statistically significant decrease in use of low-value cancer screening composite among individuals enrolled in TM and those enrolled in MA from the 2006 to 2008 period to the 2014 to 2015 period (OR, 0.29; 95% CI, 0.11-0.77; *P* = .01) (Table 4), but there were no differences in the low-value cancer screening composite measure between individuals enrolled in TM and those enrolled in MA over time. However, a different trend between individuals enrolled in TM and those enrolled in MA was observed in a few composites. We found that the low-value imaging composite measure was statistically significantly higher among individuals enrolled in MA compared with individuals enrolled in TM in 2006 to 2007 (OR, 1.98; 95% CI, 1.15-3.42; *P* = .01), but the trend reversed, with statistically significantly higher rates among individuals enrolled in TM in 2012 to 2013 (OR, 0.39; 95% CI, 0.18-0.85; *P* = .02) and 2014 to 2015 (OR, 0.46; 95%, CI, 0.23-0.96; P = .04). The numbers of individuals eligible for each measure were consistent across the 5 periods (eTable 5 in the Supplement). Although the numbers of individuals eligible for each measure were higher in 2014 to 2015, this may be associated with a larger sample size in that period (eTable 6 in the Supplement).

**Figure.**
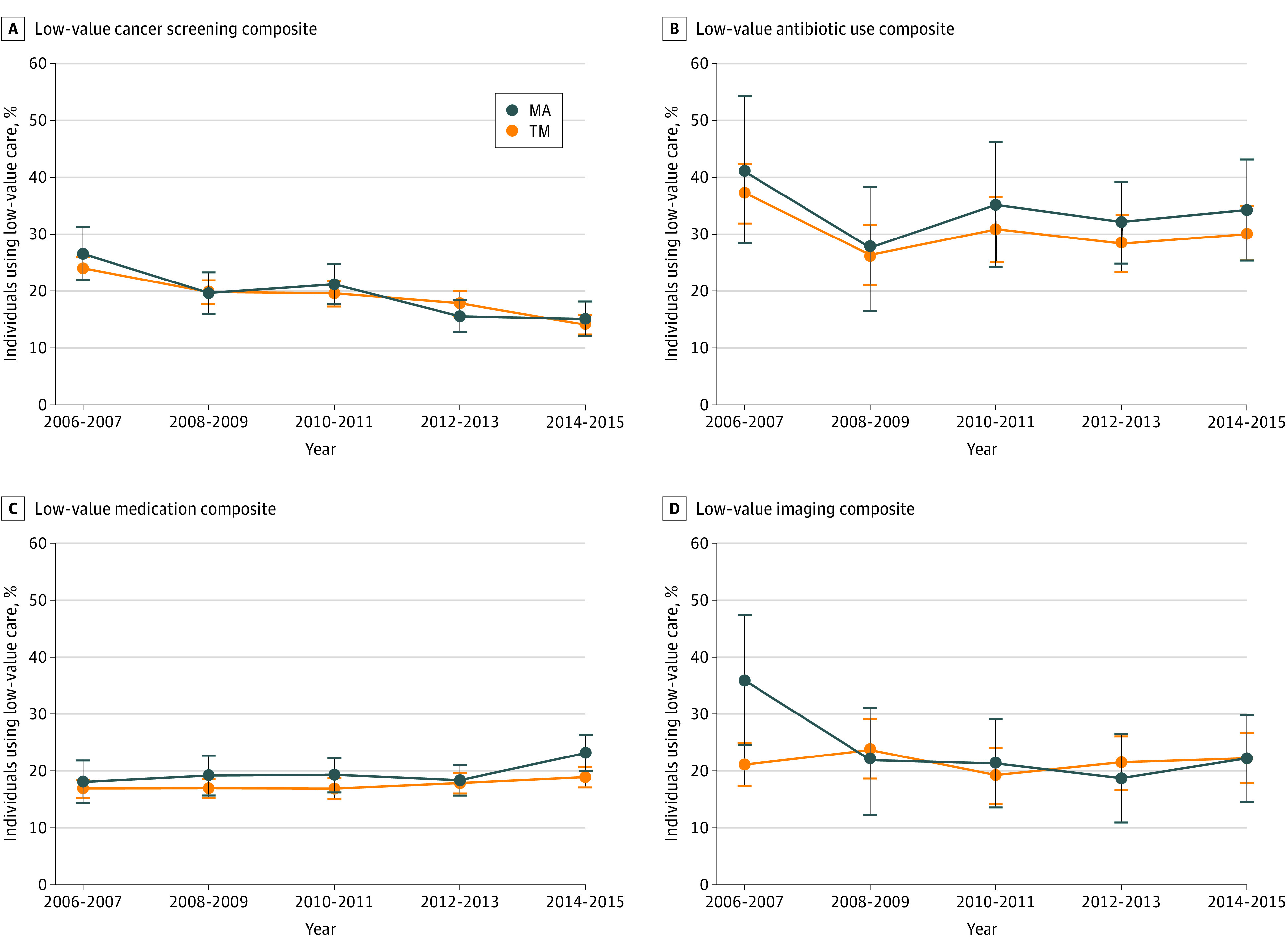
Trends in Use of Low-Value Care From 2006 to 2015 Error bars indicate 95% CIs; TM, traditional Medicare; MA, Medicare Advantage.

**Table 4.** Logistic Regression for Use of Low-Value Care, 2006 to 2015

Variable	Low-value care composite							
	Cancer screening		Antibiotic use		Medication		Imaging	
	OR (95% CI)^a^	*P* value	OR (95% CI)^a^	*P* value	OR (95% CI)^a^	*P* value	OR (95% CI)^a^	*P* value
MA enrollment	1.16 (0.86-1.55)	.33	1.15 (0.62-2.14)	.66	1.11 (0.82-1.51)	.48	1.98 (1.15-3.42)	.01
Period								
2006-2007	1 [Reference]	NA	1 [Reference]	NA	1 [Reference]	NA	1 [Reference]	NA
2008-2009	0.78 (0.41-1.50)	.46	0.45 (0.13-1.62)	.22	0.70 (0.38-1.29)	.25	1.58 (0.41-6.12)	.51
2010-2011	0.55 (0.25-1.18)	.13	0.20 (0.05-0.88)	.03	0.75 (0.38-1.48)	.40	1.24 (0.31-4.95)	.76
2012-2013	0.44 (0.19-1.01)	.05	0.32 (0.07-1.40)	.13	1.04 (0.49-2.19)	.93	1.52 (0.32-7.15)	.59
2014-2015	0.29 (0.11-0.77)	.01	0.33 (0.06-1.70)	.18	0.98 (0.43-2.25)	.96	1.14 (0.20-6.68)	.88
Interaction between MA and period								
MA × 2006-2007	1 [Reference]	NA	1 [Reference]	NA	1 [Reference]	NA	1 [Reference]	NA
MA × 2008-2009	0.88 (0.58-1.35)	.57	0.94 (0.39-2.24)	.89	1.07 (0.72-1.61)	.73	0.47 (0.20-1.08)	.08
MA × 2010-2011	0.95 (0.63-1.42)	.79	0.90 (0.40-2.06)	.81	1.07 (0.72-1.58)	.75	0.58 (0.26-1.30)	.19
MA × 2012-2013	0.72 (0.48-1.10)	.13	1.03 (0.49-2.18)	.94	0.93 (0.63-1.36)	.70	0.39 (0.18-0.85)	.02
MA × 2014-2015	0.95 (0.62-1.45)	.80	0.93 (0.43-2.00)	.86	1.19 (0.82-1.74)	.37	0.46 (0.23-0.96)	.04

Abbreviations: MA, Medicare Advantage; NA, not applicable; OR, odds ratio.

^a^ Findings for other covariates are not shown.

## Discussion

This cross-sectional study had 2 key findings on use of low-value care among individuals enrolled in TM and MA. First, few differences were found overall in use of low-value care between TM and MA populations. Statistically significant differences were found in the low-value medication composite and the NSAID use in hypertension, heart failure, or kidney disease measure. Second, there was little evidence for decreases in use of low-value care in TM and MA over time, with a decrease found in 1 measure.

We found that use of low-value care in MA was as prevalent as in TM during the study period, suggesting that the structure of MA may not be associated with decreases in use of low-value care. These findings may appear to contradict results of studies from 2017 to 2020,^16,17,18,19,20^ which found that MA provided lower rates of unnecessary care and intensive care than TM. There are several potential explanations for our findings. First, a 2017 study^41^ found that a large share of low-value care was low cost or very low cost; MA plans may not be motivated to reduce low-cost services given that the potential for cost saving from low-cost services may be modest. Second, MA plans may promote targeted investment in areas that are associated with quality score or payment levels, but low-value care metrics may not be targeted as part of these scores or payments. Third, there may still be misaligned incentives for clinicians in MA plans to decrease provision of low-value care. Some clinicians serving individuals enrolled in MA are still operating under volume-based incentives, which are associated with less leverage in decreasing low-value care. Use of low-value services in our study was higher among individuals enrolled in MA compared with those enrolled in TM in almost all services, although the differences were statistically insignificant for most services. These findings suggest the need to reconsider the design of financial incentives in the TM and MA programs.

Our null findings for decreases in use of low-value care in TM or MA over time are consistent with the results of a 2016 study.^25^ This may suggest that recent efforts aimed at decreasing use of low-value care have a limited role and are associated with a limited outcome. However, 2 other findings in our study were notable. First, decreasing rates in the low-value cancer screening composite measure in both programs were of interest. This finding may suggest that it takes time to observe significant changes, owing to low participation rates in early years and time lags in changes in practice patterns. Second, individuals enrolled in MA had a higher likelihood of having a low-value imaging composite use compared with individuals enrolled in TM in 2006 to 2007, but low-value imaging composite rates remained lower among those enrolled in MA in later years. This may suggest that decreases in use of some low-value services are partly associated with MA’s more recent efforts to decrease low-value imaging services that are costly.^2^

### Limitations

This study has several limitations. First, we could not measure all potentially relevant exclusions when identifying the use of low-value care. The MEPS reports health conditions based on CCS or 3-digit *ICD-9-CM* diagnosis and procedure codes, and thus we could not precisely identify individuals with competing diagnoses or exclude all conditions associated with clinical red flags. Our identification of low-value service use might thus have included some inappropriate individuals and conditions. Second, there may be selection into MA. Although we accounted for differences in sample characteristics between individuals enrolled in TM and those enrolled in MA, unobserved differences in patient factors may have remained. Third, our measure of cancer screening was self-reported and thus may be subject to reporting bias. Fourth, the MEPS reports information on medical conditions, but there may be reporting bias because individuals were not necessarily required to self-report all information and the MEPS does not verify self-reported information. Fifth, we detected statistically insignificant differences in use of some low-value care between TM and MA populations, but this might be associated with the small sample size.

## Conclusions

This cross-sectional study found that there were no significant differences in most measures of use of low-value care between individuals enrolled in TM and those enrolled in MA from 2006 to 2015. These findings suggest that neither the current structure of the TM and MA programs nor recent efforts to decrease use of low-value care have been associated with significant outcomes.

## Notes

Supplement. **eTable 1.** Logistic Regression to Estimate Inverse Probability of Treatment Weighting as a Propensity for Enrolling in Medicare Advantage Based on Control Variables **eTable 2.** Sample Characteristics by Period, 2006 to 2015 **eTable 3.** Odds Ratios From Logistic Regression of Use of Low-Value Care, 2006 to 2015 **eTable 4.** Subgroup Analyses of Use of Low-Value Care, 2006 to 2015 **eTable 5.** Descriptive Statistics of Outcomes by Period, 2006 to 2015 **eTable 6.** Sample Size by Year **eFigure.** Propensity Scores Determination
