# Supplementary material for: Trends in Use of Low-Value Care in Traditional Fee-for-Service Medicare and Medicare Advantage
Source: JAMA Netw Open. 2021 Mar 17;4(3):e211762. doi: 10.1001/jamanetworkopen.2021.1762 (PMC7970337; doi:10.1001/jamanetworkopen.2021.1762)
Supplement: Supplement. — eTable 1. Logistic Regression to Estimate Inverse Probability of Treatment Weighting as a Propensity for Enrolling in Medicare Advantage Based on Control Variables eTable 2. Sample Characteristics by Period, 2006 to 2015 eTable 3. Odds Ratios From Logistic Regression of Use of Low-Value Care, 2006 to 2015 eTable 4. Subgroup Analyses of Use of Low-Value Care, 2006 to 2015 eTable 5. Descriptive Statistics of Outcomes by Period, 2006 to 2015 eTable 6. Sample Size by Year eFigure. Propensity Scores Determination [file jamanetwopen-e211762-s001.pdf]

Supplemental Online Content

Park S, Jung J, Burke RE, Larson EB. Trends in use of low-value care in traditional fee-for-service Medicare and Medicare Advantage. *JAMA Netw Open*. 2021;4(3):e211762. doi:10.1001/jamanetworkopen.2021.1762

eTable 1. Logistic Regression to Estimate Inverse Probability of Treatment Weighting as a Propensity for Enrolling in Medicare Advantage Based on Control Variables
eTable 2. Sample Characteristics by Period, 2006 to 2015
eTable 3. Odds Ratios From Logistic Regression of Use of Low-Value Care, 2006 to 2015
eTable 4. Subgroup Analyses of Use of Low-Value Care, 2006 to 2015
eTable 5. Descriptive Statistics of Outcomes by Period, 2006 to 2015
eTable 6. Sample Size by Year
eFigure. Propensity Scores Determination

This supplemental material has been provided by the authors to give readers additional information about their work.

© 2021 Park S et al. *JAMA Network Open.*

eTable 1. Logistic Regression to Estimate Inverse Probability of Treatment Weighting as a Propensity for Enrolling in Medicare Advantage Based on Control Variables

	MA enrollment														
	2006-07			2008-09			2010-11			2012-13			2014-15		
Characteristic	OR	95% CI	P value	OR	95% CI	P value	OR	95% CI	P value	OR	95% CI	P value	OR	95% CI	P value
Age															
65-69	REF			REF			REF			REF			REF		
70-74	0.96	0.71 to 1.30	0.800	0.77	0.59 to 1.01	0.055	0.91	0.70 to 1.17	0.459	0.91	0.72 to 1.15	0.441	1.47	1.17 to 1.85	0.001
75-79	1.10	0.80 to 1.51	0.545	0.88	0.65 to 1.20	0.426	1.08	0.83 to 1.43	0.559	1.14	0.89 to 1.47	0.300	1.07	0.83 to 1.38	0.599
>=80	1.05	0.77 to 1.44	0.743	0.94	0.70 to 1.25	0.653	0.96	0.73 to 1.26	0.769	1.13	0.89 to 1.45	0.318	0.96	0.73 to 1.26	0.767
Female	1.17	0.93 to 1.47	0.182	1.21	0.98 to 1.50	0.073	1.03	0.85 to 1.25	0.764	1.16	0.96 to 1.40	0.125	1.13	0.94 to 1.35	0.203
Race/ethnicity															
Non-Hispanic white	REF			REF			REF								
Hispanic	3.55	2.25 to 5.61	<.001	3.60	2.50 to 5.18	<.001	2.13	1.51 to 2.99	<.001	2.70	1.99 to 3.66	<.001	2.68	1.97 to 3.63	<.001
Non-Hispanic black	1.22	0.84 to 1.78	0.300	1.75	1.30 to 2.35	<.001	1.46	1.11 to 1.91	0.006	1.26	0.99 to 1.60	0.062	1.28	1.00 to 1.65	0.052
Non-Hispanic Asian	1.59	0.97 to 2.60	0.066	2.21	1.38 to 3.55	0.001	1.36	0.83 to 2.24	0.222	1.37	0.94 to 2.00	0.099	1.84	1.26 to 2.71	0.002
Non-Hispanic other or multiple	1.86	0.82 to 4.23	0.139	0.31	0.13 to 0.73	0.007	1.20	0.58 to 2.50	0.623	0.58	0.25 to 1.35	0.203	0.27	0.14 to 0.52	<.001
Married	1.08	0.85 to 1.37	0.528	0.89	0.72 to 1.10	0.275	0.91	0.75 to 1.11	0.366	1.01	0.83 to 1.22	0.945	1.14	0.94 to 1.38	0.198
Family income															
<200% FPL	REF			REF			REF								
200-399% FPL	1.09	0.83 to 1.43	0.551	1.21	0.95 to 1.54	0.12	1.01	0.81 to 1.26	0.918	1.00	0.80 to 1.24	0.965	0.85	0.68 to 1.07	0.177
>=400% FPL	0.81	0.62 to 1.07	0.138	0.92	0.72 to 1.18	0.52	0.71	0.57 to 0.90	0.004	0.60	0.48 to 0.76	<.001	0.55	0.43 to 0.69	<.001
US census regions															
Northeast	REF			REF			REF								
Midwest	0.80	0.55 to 1.16	0.229	0.69	0.50 to 0.94	0.018	0.59	0.44 to 0.79	<.001	0.65	0.49 to 0.86	0.002	0.70	0.52 to 0.93	0.016
South	0.79	0.56 to 1.10	0.165	0.70	0.53 to 0.94	0.017	0.55	0.42 to 0.73	<.001	0.65	0.50 to 0.84	0.001	0.78	0.60 to 1.01	0.060
West	5.83	4.14 to 8.21	<.001	3.08	2.27 to 4.17	<.001	1.78	1.33 to 2.39	<.001	1.63	1.24 to 2.16	0.001	1.44	1.08 to 1.91	0.013
Perceived health status															
Excellent, very good, or good	REF			REF			REF								
Poor or fair	0.78	0.57 to 1.06	0.112	0.80	0.61 to 1.06	0.125	1.01	0.77 to 1.32	0.967	0.97	0.75 to 1.26	0.81	0.99	0.76 to 1.29	0.924
Perceived mental health status															
Excellent, very good, or good	REF			REF			REF								
Poor or fair	0.93	0.61 to 1.42	0.733	1.16	0.78 to 1.73	0.461	1.04	0.73 to 1.48	0.838	1.01	0.72 to 1.42	0.945	0.69	0.49 to 0.98	0.036
Limitation															
Any	0.80	0.57 to 1.14	0.223	1.03	0.75 to 1.41	0.85	1.06	0.78 to 1.45	0.712	1.25	0.92 to 1.69	0.148	0.93	0.72 to 1.20	0.568
Cognitive	0.99	0.69 to 1.42	0.962	1.05	0.73 to 1.52	0.776	0.96	0.70 to 1.32	0.82	0.93	0.69 to 1.26	0.64	1.11	0.83 to 1.49	0.491

© 2021 Park S et al. *JAMA Network Open.*

Functional	1.23	0.92 to 1.64	0.158	0.84	0.64 to 1.09	0.195	1.10	0.86 to 1.40	0.46	0.82	0.64 to 1.04	0.106	0.81	0.64 to 1.02	0.069
Social	0.87	0.60 to 1.28	0.492	1.08	0.77 to 1.52	0.648	0.83	0.59 to 1.17	0.295	0.95	0.70 to 1.29	0.748	1.19	0.89 to 1.59	0.248
Chronic conditions															
Angina	1.56	1.00 to 2.42	0.051	0.69	0.46 to 1.04	0.076	1.10	0.76 to 1.61	0.611	0.83	0.58 to 1.20	0.326	0.77	0.53 to 1.13	0.178
Arthritis	1.23	0.97 to 1.55	0.087	1.22	0.98 to 1.52	0.073	0.89	0.73 to 1.09	0.263	1.09	0.91 to 1.32	0.349	1.18	0.97 to 1.44	0.100
Asthma	1.20	0.83 to 1.71	0.331	0.63	0.44 to 0.91	0.015	0.76	0.53 to 1.09	0.142	0.77	0.57 to 1.05	0.102	1.21	0.87 to 1.68	0.268
Coronary heart disease	0.97	0.67 to 1.40	0.877	0.96	0.70 to 1.33	0.826	1.02	0.76 to 1.37	0.888	1.07	0.81 to 1.41	0.645	1.10	0.84 to 1.44	0.470
Diabetes	0.79	0.63 to 1.00	0.045	1.13	0.91 to 1.41	0.254	0.99	0.81 to 1.20	0.902	1.16	0.96 to 1.41	0.117	1.28	1.04 to 1.56	0.018
Emphysema	1.11	0.84 to 1.47	0.470	1.18	0.91 to 1.52	0.205	0.85	0.68 to 1.08	0.193	1.14	0.92 to 1.42	0.219	1.01	0.81 to 1.25	0.941
High cholesterol	0.69	0.38 to 1.25	0.221	1.36	0.92 to 2.02	0.128	1.13	0.77 to 1.64	0.531	1.12	0.78 to 1.60	0.553	0.98	0.68 to 1.42	0.905
High blood pressure	0.94	0.74 to 1.18	0.583	1.02	0.81 to 1.29	0.853	1.14	0.92 to 1.41	0.239	1.17	0.95 to 1.43	0.134	0.81	0.66 to 0.99	0.045
MI	0.96	0.63 to 1.48	0.866	1.21	0.84 to 1.73	0.301	1.04	0.74 to 1.45	0.831	0.92	0.67 to 1.27	0.615	0.87	0.63 to 1.19	0.383
Stroke	0.96	0.73 to 1.27	0.788	0.93	0.72 to 1.20	0.568	0.93	0.74 to 1.17	0.528	0.83	0.67 to 1.02	0.075	0.91	0.74 to 1.13	0.402
Other heart conditions	1.15	0.80 to 1.65	0.450	0.69	0.50 to 0.95	0.022	1.09	0.81 to 1.45	0.585	1.03	0.77 to 1.37	0.841	1.14	0.87 to 1.50	0.354
Cancer	0.71	0.52 to 0.96	0.026	1.00	0.79 to 1.28	0.991	0.84	0.67 to 1.04	0.112	0.84	0.68 to 1.02	0.084	0.90	0.73 to 1.12	0.343
ADRD	0.64	0.34 to 1.21	0.173	0.45	0.23 to 0.88	0.019	0.96	0.58 to 1.57	0.863	1.26	0.72 to 2.19	0.420	0.76	0.43 to 1.35	0.352
Year															
2006	REF														
2007	1.17	0.95 to 1.45	0.143												
2008				REF											
2009				1.50	1.24 to 1.83	0.055									
2010							REF								
2011							1.06	0.89 to 1.27	0.51						
2012										REF					
2013										1.23	1.04 to 1.46	0.016			
2014													REF		
2015													0.84	0.7 to 1	0.049

Abbreviations: TM, traditional Medicare; MA, Medicare Advantage; FPL, federal poverty level; MI, myocardial infarction; ADRD, Alzheimer's disease and related dementias.

© 2021 Park S et al. *JAMA Network Open.*

eTable 2. Sample Characteristics by Period, 2006 to 2015

	Weighted % with IPTW[a]									
	2006-07		2008-09		2010-11		2012-13		2014-15	
Characteristic	TM	MA	TM	MA	TM	MA	TM	MA	TM	MA
Age										
65-69	26.0	27.1	24.4	25.3	28.0	28.4	30.4	30.4	29.4	29.1
70-74	23.6	22.7	25.0	23.4	26.6	26.6	27.0	27.2	25.9	25.9
75-79	23.0	23.5	21.0	22.4	19.6	19.7	18.7	18.4	18.8	18.5
>=80	27.5	26.6	29.6	28.8	25.8	25.3	23.9	24.0	25.9	26.5
Female	56.6	54.6	56.7	57.0	56.2	56.0	53.8	53.5	54.8	55.3
Race/ethnicity										
Non-Hispanic white	86.7	87.1	85.0	85.2	85.4	85.4	84.4	84.7	83.6	83.1
Hispanic	4.3	4.0	5.6	5.3	4.7	4.6	5.0	4.9	4.6	4.6
Non-Hispanic black	6.1	6.3	5.7	5.8	6.7	6.7	6.9	6.8	6.6	6.7
Non-Hispanic Asian	1.8	1.4	2.5	2.5	2.2	2.3	2.2	2.1	2.9	2.9
Non-Hispanic other or multiple	1.1	1.1	1.2	1.2	1.1	1.0	1.5	1.5	2.4	2.6
Married	58.2	58.6	58.2	58.7	58.9	59.0	59.0	58.9	59.5	59.5
Family income										
<200% FPL	30.9	30.5	29.6	29.3	28.0	27.3	27.9	27.7	26.5	27.0
200-399% FPL	31.1	34.7	31.5	32.1	30.8	30.8	30.2	30.4	27.6	28.0
>=400% FPL	38.0	34.8	38.9	38.6	41.2	41.9	41.9	41.9	45.9	45.0
US census regions										
Northeast	18.3	18.3	17.7	16.4	17.7	17.4	16.0	15.9	17.2	17.5
Midwest	22.9	22.7	23.4	24.4	25.6	26.1	25.3	26.2	23.1	22.9
South	40.4	40.9	39.8	40.1	36.0	35.8	37.8	37.1	38.4	38.5
West	18.5	18.2	19.1	19.1	20.7	20.6	20.9	20.8	21.2	21.1
Perceived health status										
Excellent, very good, or good	78.4	78.5	78.8	78.8	81.7	81.8	82.9	83.8	81.6	81.8
Poor or fair	21.6	21.3	21.1	21.0	18.0	18.1	16.9	16.1	18.0	18.2
Perceived mental health status										
Excellent, very good, or good	89.8	89.9	91.5	91.3	91.1	91.1	91.8	92.1	90.3	90.9
Poor or fair	10.2	9.8	8.3	8.6	8.7	8.8	8.0	7.8	9.4	9.1
Limitation										
Any	28.1	26.7	27.1	27.0	24.4	24.3	26.4	26.3	27.5	27.1
Functional	13.8	12.2	13.3	14.0	11.4	10.8	13.9	13.2	14.0	14.0
Cognitive	43.5	43.8	42.8	43.7	40.7	40.7	39.9	39.3	42.1	41.5
Social	17.0	17.5	15.2	16.1	14.1	13.8	14.9	14.8	16.4	16.4
Chronic conditions										
Angina	8.1	7.7	9.3	9.2	8.1	8.0	7.5	7.3	7.9	8.3
Arthritis	54.7	55.7	61.3	60.5	60.6	59.7	59.8	60.1	60.6	61.1
Asthma	10.0	8.5	7.2	6.9	8.5	8.6	8.6	8.3	9.0	10.1
Coronary heart disease	15.1	16.6	22.5	21.7	20.6	20.7	20.3	20.0	19.8	19.8
Diabetes	19.2	18.7	20.9	21.4	19.8	19.4	21.6	21.8	22.7	22.8
Emphysema	5.3	5.8	7.2	7.7	7.1	6.8	6.7	6.9	6.4	6.3
High cholesterol	57.4	57.9	63.2	63.4	64.0	64.2	65.4	65.2	65.4	65.8
High blood pressure	65.0	64.6	68.3	68.1	70.0	69.5	69.9	69.5	68.6	68.2
MI	10.8	11.0	13.8	14.4	11.4	11.6	12.4	12.2	12.4	12.3
Stroke	19.9	20.2	30.5	30.3	28.8	28.4	29.2	28.7	32.3	31.8
Other heart conditions	9.5	8.3	13.2	12.3	12.7	12.5	11.9	12.1	11.2	11.3
Cancer	19.4	22.6	25.4	24.5	24.6	24.5	23.4	23.0	25.6	26.1
ADRD	3.9	3.5	3.3	3.0	3.4	3.3	3.1	3.0	3.4	3.6

Abbreviations: TM, traditional Medicare; MA, Medicare Advantage; IPTW, inverse probability of treatment weighting; FPL, federal poverty level; MI, myocardial infarction; ADRD, Alzheimer's disease and related dementias.

[a] We used the IPTW estimated for each time period (2006-07, 2008-09, 2010-11, 2012-13, and 2014-15).

© 2021 Park S et al. *JAMA Network Open.*

eTable 3. Odds Ratios From Logistic Regression of Use of Low-Value Care, 2006 to 2015

Measure or composite	Odds ratio of receiving low-value care among MA enrollees relative to TM enrollees (95% CI)[a]
Low-value cancer screening composite	1.1 (0.8 to 1.5)
Cervical cancer screening	1.1 (0.7 to 1.6)
Colorectal cancer screening	0.6 (0.3 to 1.2)
Prostate cancer screening	1.8 (1.1 to 3.1)
Low-value antibiotic use composite	1.1 (0.6 to 2.1)
Antibiotics for acute upper respiratory infection	1.3 (0.6 to 2.4)
Antibiotics for influenza	0.2 (0.0 to 1.7)
Low-value medications composite	1.1 (0.8 to 1.5)
Anxiolytics, sedatives, and hypnotics	0.8 (0.5 to 1.1)
Benzodiazepine for depression	0.6 (0.3 to 1.4)
Opioid for headache	NA[b]
Opioid for back pain	1.5 (0.7 to 3.0)
NSAID use for hypertension, heart failure, or kidney disease	1.6 (1.0 to 2.6)
Low-value imaging composite	1.9 (1.1 to 3.4)
MRI/CT for back pain	1.0 (0.5 to 2.1)
Radiograph for back pain	2.0 (1.1 to 3.8)
MRI/CT for headache	NA[b]

Abbreviations: TM, traditional Medicare; MA, Medicare Advantage; NSAID, nonsteroidal anti-inflammatory drug; CT, computed tomography; MRI, magnetic resonance imaging.

[a] To account for differences in characteristics between TM and MA enrollees attributable to selection bias, we computed the inverse probability of treatment weighting (IPTW) as a propensity for enrolling in MA based on enrollee demographic, socioeconomic, and health status variables. To account for changes in the composition of TM and MA enrollees over time, we estimated the IPTW separately for each of the following four time periods (2006-07, 2008-09, 2010-11, 2012-13, and 2014-15). We then performed a logistic regression model after controlling all covariates described above as well as MA enrollment, time period (2006-07, 2008-09, 2010-11, 2012-13, and 2014-15), the interaction term between MA enrollment and time period, the interaction term between US census regions (Northeast, Midwest, South, and West) and time period, and the share of Medicare beneficiaries enrolled in MA plans by year and US census region and applied the IPTW. Findings for other covariates were not shown.

[b] There were less than five enrollees with use of low-value care.

© 2021 Park S et al. *JAMA Network Open.*

eTable 4. Subgroup Analyses of Use of Low-Value Care, 2006 to 2015

Measure or composite	Difference in use of low-value care among MA enrollees relative to TM enrollees, mean % (95% CI)[a]				
	Non-Hispanic white	Good perceived health (including excellent, very good, or good)	Poor perceived health (including poor or fair)	Family income less than 400% FPL	Family income higher than 400% FPL
Low-value cancer screening composite	0.2 (-1.9 to 2.4)	0.8 (-1.4 to 2.9)	-0.2 (-4.2 to 3.8)	-0.1 (-2.2 to 2.0)	1.7 (-1.9 to 5.3)
Cervical cancer screening	0.7 (-1.4 to 2.9)	0.1 (-2.0 to 2.2)	2.1 (-1.5 to 5.8)	-0.6 (-2.6 to 1.4)	3.3 (-0.5 to 7.1)
Colorectal cancer screening	-1.2 (-2.8 to 0.4)	-0.7 (-2.2 to 0.8)	-1.7 (-5.4 to 2.1)	-0.9 (-2.6 to 0.7)	-1.2 (-4.0 to 1.7)
Prostate cancer screening	0.2 (-4.5 to 4.8)	2.6 (-2.4 to 7.7)	-5.2 (-11.9 to 1.4)	2.4 (-2.6 to 7.4)	-2.8 (-10 to 4.5)
Low-value antibiotic use composite	1.5 (-3.8 to 6.8)	2.2 (-3.0 to 7.4)	3.4 (-6.7 to 13.5)	2.0 (-4.0 to 8.0)	4.7 (-2.6 to 12.1)
Antibiotics for acute upper respiratory infection	2.0 (-3.9 to 7.8)	2.2 (-3.4 to 7.9)	7.6 (-3.3 to 18.4)	2.8 (-3.8 to 9.4)	4.4 (-3.6 to 12.3)
Antibiotics for influenza	1.5 (-7.3 to 10.3)	3.0 (-5.3 to 11.4)	0.0 (0.0 to 0.0)	5.6 (-3.9 to 15.0)	0.0 (0.0 to 0.0)
Low-value medications composite	2.7 (0.8 to 4.5)	1.8 (0.1 to 3.5)	4.2 (-0.2 to 8.5)	2.2 (0.2 to 4.3)	2.2 (-0.4 to 4.9)
Anxiolytics, sedatives, and hypnotics	0.7 (-0.7 to 2.1)	0.8 (-0.5 to 2.1)	0.5 (-2.5 to 3.4)	0.1 (-1.4 to 1.6)	1.6 (-0.4 to 3.5)
Benzodiazepine for depression	2.7 (-4.0 to 9.3)	-1.0 (-8.2 to 6.1)	1.3 (-8.7 to 11.4)	-1.5 (-8.4 to 5.5)	7.3 (-3.2 to 17.7)
Opioid for headache	NA[b]	NA[b]	NA[b]	NA[b]	NA[b]
Opioid for back pain	1.0 (-2.7 to 4.6)	0.2 (-3.2 to 3.5)	4.4 (-3.8 to 12.6)	6.2 (1.2 to 11.3)	-4.5 (-8.8 to -0.3)
NSAID use for hypertension, heart failure, or kidney disease	3.4 (1.5 to 5.3)	2.4 (0.6 to 4.1)	5.2 (1.1 to 9.2)	2.7 (0.7 to 4.7)	2.5 (-0.2 to 5.3)
Low-value imaging composite	2.3 (-2.3 to 7.0)	-0.5 (-5.3 to 4.4)	9.7 (1.5 to 17.9)	3.3 (-2.0 to 8.6)	-1.3 (-7.9 to 5.2)
MRI/CT for back pain	1.2 (-2.4 to 4.9)	-1.1 (-4.9 to 2.7)	6.7 (-0.8 to 14.2)	1.1 (-2.8 to 4.9)	0.5 (-5.1 to 6.0)
Radiograph for back pain	2.7 (-1.8 to 7.2)	1.7 (-2.9 to 6.3)	0.5 (-5.6 to 6.7)	3.2 (-1.9 to 8.3)	-0.1 (-5.9 to 5.7)
MRI/CT for headache	NA[b]	NA[b]	NA[b]	NA[b]	NA[b]

Abbreviations: TM, traditional Medicare; MA, Medicare Advantage; FPL, federal poverty level; NSAID, nonsteroidal anti-inflammatory drug; CT, computed tomography; MRI, magnetic resonance imaging.

[a] To account for differences in characteristics between TM and MA enrollees attributable to selection bias, we computed the IPTW as a propensity for enrolling in MA based on enrollee demographic, socioeconomic, and health status variables. To account for changes in the composition of TM and MA enrollees over time, we estimated the IPTW separately for each of the following four time periods (2006-07, 2008-09, 2010-11, 2012-13, and 2014-15). We then performed a logistic regression model after controlling demographic socioeconomic variables as well as MA enrollment, time period (2006-07, 2008-09, 2010-11, 2012-13, and 2014-15), the interaction term between MA enrollment and time period, the interaction term between time period and US census regions, and census-level MA penetration and applied the IPTW. Next, we estimated the mean adjusted values of the outcomes for TM and MA enrollees. We examined the difference in these adjusted mean outcomes among MA enrollees relative to TM enrollees.

[b] There were less than five enrollees with use of low-value care.

© 2021 Park S et al. *JAMA Network Open.*

eTable 5. Descriptive Statistics of Outcomes by Period, 2006 to 2015

	2006-07				2008-09				2010-11				2012-13				2014-15			
Outcome	TM	%	MA	%	TM	%	MA	%	TM	%	MA	%	TM	%	MA	%	TM	%	MA	%
Low-value cancer screening composite	2048	22.7	510	23.5	1654	19.5	670	18.8	1534	18.7	772	16.7	1416	17.9	1004	15.9	1723	14.5	837	14.8
Cervical cancer screening	1532	16.3	721	18.0	1222	14.0	520	13.1	1188	13.0	596	10.4	1086	14.1	794	12.1	1279	9.8	661	11.8
Colorectal cancer screening	1230	8.9	461	6.8	1004	6.8	382	5.5	853	6.9	435	6.9	743	4.7	537	3.7	1000	4.0	437	4.1
Prostate cancer screening	516	30.8	173	34.6	432	27.5	150	28.7	346	29.5	176	29.5	330	24.2	210	25.2	444	23.6	176	21.0
Low-value antibiotic use composite	397	38.3	228	34.3	335	26.0	130	22.3	333	30.6	127	30.7	406	31.0	260	26.9	471	32.5	183	32.8
Antibiotics for acute upper respiratory infection	366	39.1	169	38.6	300	26.7	120	22.5	310	31.0	115	31.3	301	35.2	200	29.5	381	34.6	140	37.9
Antibiotics for influenza	39	25.6	65	11.1	44	18.2	12	25.0	29	20.7	14	21.4	126	17.5	71	15.5	101	20.8	52	15.4
Low-value medications composite	2799	16.7	1257	18.6	2249	17.9	873	19.0	2149	16.7	1055	18.5	2068	17.9	1387	18.5	2371	19.2	1175	20.9
Anxiolytics, sedatives, and hypnotics	2757	8.2	1230	8.7	2223	8.5	865	8.7	2121	8.8	1039	8.6	2021	8.5	1361	8.4	2339	7.9	1154	8.5
Benzodiazepine for depression	237	43.5	129	49.2	198	44.4	63	42.9	197	43.1	78	47.4	217	51.2	134	47.0	278	45.0	119	46.2
Opioid for headache	92	0.0	50	0.0	43	0.0	18	0.0	53	0.0	27	0.0	73	0.0	44	0.0	70	0.0	36	0.0
Opioid for back pain	493	15.2	202	21.3	300	17.3	126	18.3	287	12.2	138	16.7	346	11.8	185	13.5	421	14.5	205	13.7
NSAID use for hypertension, heart failure, or kidney disease	1724	9.7	877	11.6	1543	10.1	598	12.9	1509	9.3	748	11.8	1395	10.4	1017	11.3	1661	11.9	781	15.1
Low-value imaging composite	566	21.9	242	27.5	334	24.3	142	16.9	330	18.5	157	21.7	396	21.0	218	16.1	468	23.1	233	20.6
MRI/CT for back pain	493	13.0	202	14.8	300	12.3	126	10.3	287	8.7	138	12.3	346	11.8	185	7.6	421	13.5	205	11.2
Radiograph for back pain	493	15.0	202	20.4	300	17.0	126	15.1	287	12.5	138	13.8	346	15.3	185	11.9	421	16.6	205	13.2
MRI/CT for headache	92	13.0	50	13.3	43	9.3	18	0.0	53	13.2	27	14.8	73	4.1	44	9.1	70	8.6	36	11.1

Abbreviations: TM, traditional Medicare; MA, Medicare Advantage; NSAID, nonsteroidal anti-inflammatory drug; CT, computed tomography; MRI, magnetic resonance imaging.

© 2021 Park S et al. *JAMA Network Open.*

eTable 6. Sample Size by Year

Year	N	
	TM	MA
2006	1481	326
2007	1322	326
2008	1138	363
2009	1116	516
2010	1049	498
2011	1109	563
2012	1145	722
2013	934	672
2014	1108	589
2015	1275	589
Total	11677	5164

Abbreviations: TM, traditional Medicare; MA, Medicare Advantage.

© 2021 Park S et al. *JAMA Network Open.*

eFigure. Propensity Scores Determination

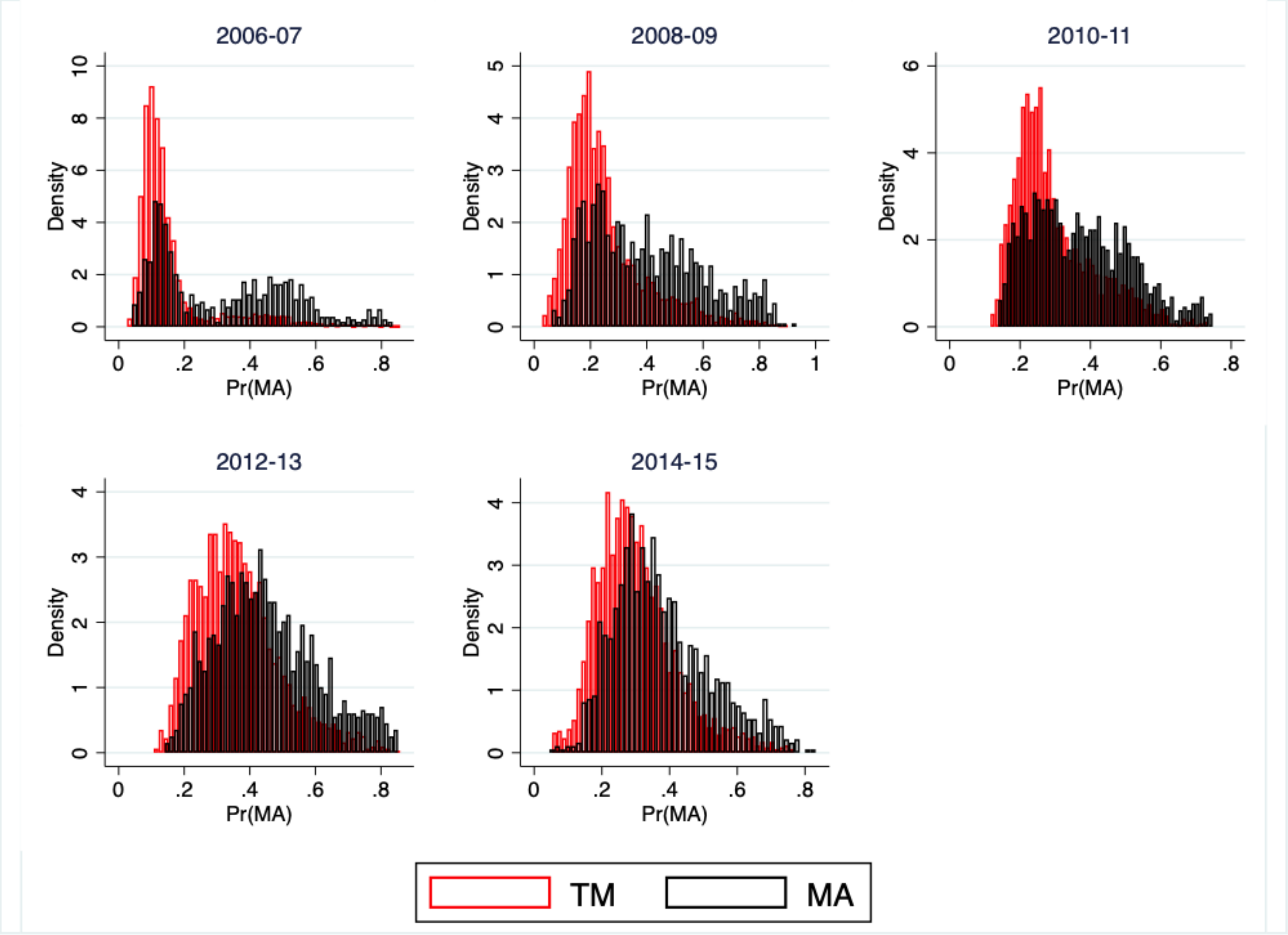

Abbreviations: TM, traditional Medicare; MA, Medicare Advantage.

© 2021 Park S et al. *JAMA Network Open.*
